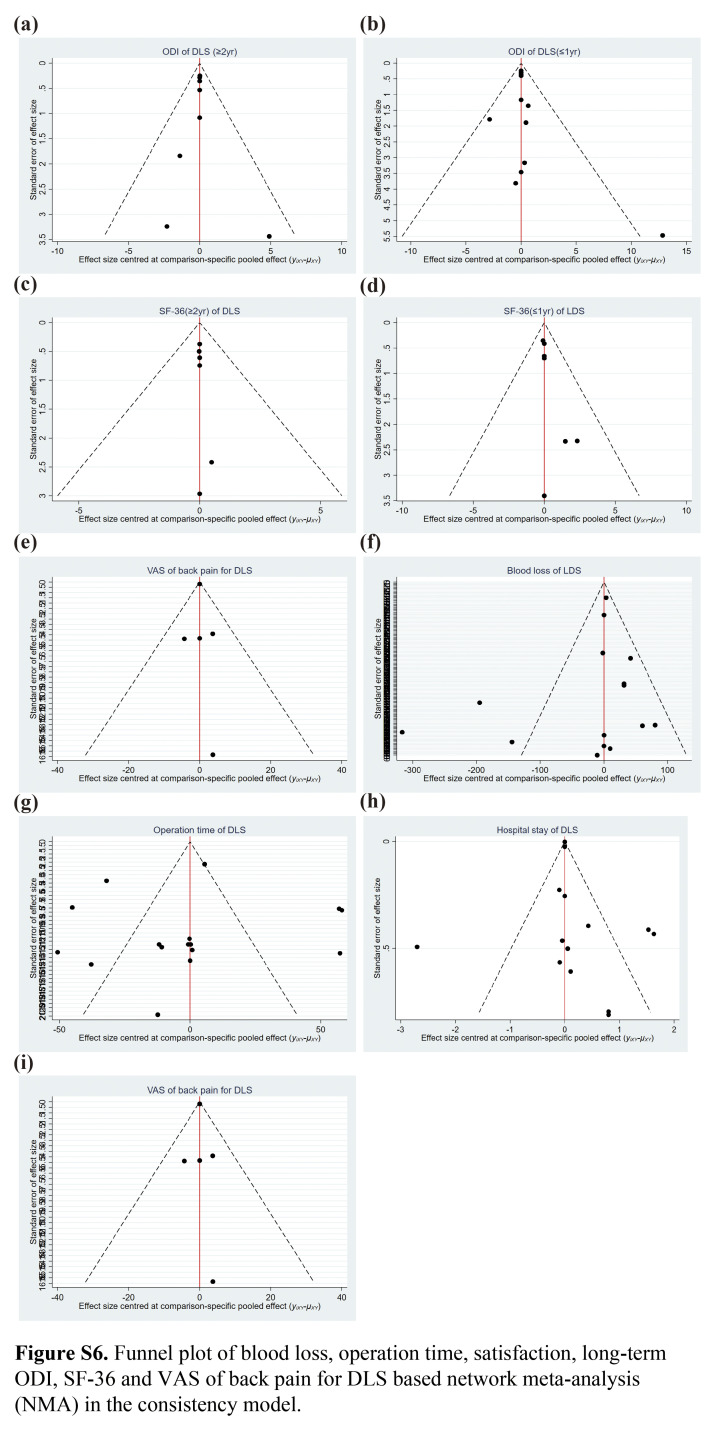# Management for degenerative lumbar spondylolisthesis: a network meta-analysis and systematic review basing on randomized controlled trials

**DOI:** 10.1097/JS9.0000000000001228

**Published:** 2024-03-04

**Authors:** Hao Jia, Zhuo Zhang, Jianpu Qin, Lipei Bao, Jun Ao, Hu Qian

**Affiliations:** Department of Orthopedic Surgery, Affiliated Hospital of Zunyi Medical University, Zunyi, People’s Republic of China

**Keywords:** complication, degenerative lumbar spondylolisthesis, disability, network meta-analysis, reoperation

## Abstract

**Background:**

Consensus on the various interventions for degenerative lumbar spondylolisthesis (DLS) remains unclear.

**Materials and methods:**

The authors searched PubMed, Embase, Cochrane Library, Web of Science, and major scientific websites until 01 November 2023, to screen eligible randomized controlled trials (RCTs) involving the treatment of DLS. The seven most common DLS interventions [nonsurgical (NS), decompression only (DO), decompression plus fusion without internal fixation (DF), decompression plus fusion with internal fixation (DFI), endoscopic decompression plus fusion (EDF), endoscopic decompression (ED), and circumferential fusion (360F)] were compared. The primary (pain and disability) and secondary (complications, reoperation rate, operation time, blood loss, length of hospital stay, and satisfaction) outcomes were analyzed.

**Results:**

Data involving 3273 patients in 16 RCTs comparing the efficacy of different interventions for DLS were reported. In terms of improving patient pain and dysfunction, there was a significant difference between surgical and NS. EDF showed the greatest improvement in short-term and long-term dysfunction (probability, 7.1 and 21.0%). Moreover, EDF had a higher complication rate (probability 70.8%), lower reoperation rate (probability, 20.2%), and caused greater blood loss (probability, 82.5%) than other surgical interventions. Endoscopic surgery had the shortest hospitalization time (EDF: probability, 42.6%; ED: probability, 3.9%). DF and DFI had the highest satisfaction scores.

**Conclusions:**

Despite the high complication rate of EDF, its advantages include improvement in pain, lower reoperation rate, and shorter hospitalization duration. Therefore, EDF may be a good option for patients with DLS as a less invasive surgical approach.

## Introduction

Highlights

- The best intervention for degenerative lumbar spondylolisthesis remains unclear.
- Randomized control trial-based network meta-analysis was conducted.
- Endoscopic decompression plus fusion may be a good option for patients with degenerative lumbar spondylolisthesis.

In 1955, Newman first defined degenerative lumbar spondylolisthesis (DLS) as the slippage of the superior conus relative to the inferior conus owing to degeneration without losing the vertebral arch isthmus^1^. DLS can contribute to the formation of spinal stenosis, leading to intermittent claudication in the lower back, with an incidence as high as 8.7%^2,3^. DLS predominately affects older patients aged >60 years, with a predilection for women^4,5^. Of all patients with DLS, a single spinal segment is involved in 66% of them, and two or more segments are involved in 34%, with two being the most commonly affected^3,6^. DLS presents many challenges to patients and clinicians, including an immense economic burden to society^7^.

Because of the rapid advances in medical techniques, multiple surgical and nonsurgical (NS) approaches have been developed to treat DLS. NS options include oral and injectable pain medications and physical therapy. In contrast, surgical options include decompression only (DO), decompression plus fusion without internal fixation (DF), decompression plus fusion with internal fixation (DFI), endoscopic decompression plus fusion (EDF), endoscopic decompression (ED), and circumferential fusion (360F)^2,8–11^. However, the efficacy of different treatment strategies remains controversial^3,12,13^. Therefore, providing evidence-based guidance in clinical practice is crucial. While some traditional paired meta-analyses have compared the efficacy and safety of different treatments, these studies included only two interventions, and the findings were inconsistent^12,13^. To date, no comprehensive studies involving all interventions have been reported, and the optimal intervention for DLS remains unclear.

Network meta-analyses (NMA) can be used to compare multiple interventions in a single analysis by revealing direct and indirect evidence and ranking them^14^. Therefore, the present study conducted an NMA to comprehensively compare the efficacy and safety of interventions for DLS and provide references and guidance for clinical decision-making.

## Materials and methods

### Literature search and selection

This NMA adhered to the Preferred Reporting Items for Systematic Reviews and Meta-Analyses (PRISMA, Supplemental Digital Content 1, http://links.lww.com/JS9/C45, Supplemental Digital Content 2, http://links.lww.com/JS9/C46), the PRISMA NMA extension statement, the Cochrane Collaboration recommendations, and the Assessing the methodological quality of systematic reviews (AMSTAR, Supplemental Digital Content 3, http://links.lww.com/JS9/C47) for reporting methods and results^15–18^. Related publications and abstracts comparing at least two interventions were searched in PubMed, Embase, the Cochrane Library, Web of Science, and major scientific websites before 01 November 2023. Search terms including ‘Degenerative lumbar spondylolisthesis’, ‘DLS’, ‘Lumbar spondylolisthesis’, ‘randomized controlled trial’, and ‘RCTs’, and their combinations were used in the search strategy. No restrictions were placed on the language or publication date. After the initial screening of titles and abstracts, the full-text and reference lists of relevant publications were evaluated by two independent reviewers for final inclusion. The articles’ full text was searched and evaluated when cited as potentially relevant references. The review protocol was registered in the International Prospective Register of Systematic Reviews (PROSPERO), and the registration number is CRD.

Primary eligibility criteria for the studies were as follows: a) RCTs reporting patients with DLS aged >18 years with single-segment slippage, with or without lumbar spinal stenosis; b) including the interventions mentioned above; c) reporting an outcome, including the Oswestry disability index (ODI, ranging from 0 to 100, with lower scores indicating less severe symptoms), Short Form-36 (SF-36, ranging from 0 to 100, with higher scores indicating less severe symptoms), visual analog scale (VAS), complications, reoperation, length of hospital stay, blood loss, operation time, or satisfaction. The exclusion criteria were as follows: a) Low-quality or non-RCTs, b) studies reporting none of the above results, c) follow-up duration <12 months, d) slippage after a fracture or lumbar disc herniation, and e) presence of isthmic spondylolisthesis.

### Data extraction and assessment for risk of bias

Data including the name of the first author, publication year, location of the study, intervention type, demographic characteristics (number, sex, and age), follow-up period, and clinical outcomes were independently extracted by two reviewers. Any discrepancies were resolved by consensus and arbitration through a joint manuscript review to reach an agreement. The same reviewers independently assessed the risk of bias in individual studies using the Cochrane collaboration tool (RoB2.0)^19^.

### Data synthesis and statistical analysis

In this NMA, the primary outcomes were the ODI score, SF-36, and VAS scores of back pain (continuous variables); thus, the weighted mean difference was used to present pooled estimates. The secondary outcomes were complications and reoperation (dichotomous variables). Pooled results are presented as risk ratios. Similarly, we present the 95% CIs to show the effect sizes. All statistical analyses and graphical procedures were conducted using the Stata software Version 16.0 (Stata Corp LP, United States), except for the heterogeneity test, which was performed using R software Version 4.3.1 (R Foundation for Statistical Computing). Forest plots and the *Ι* ^2^ test were conducted to assess the potential heterogeneity (values <25%, 25–75%, and >75% for the *I* ^2^ statistic represented mild, moderate, and severe heterogeneity, respectively). Inconsistency was assessed by comparing the statistics of the bias information criterion in the fitted consistency and inconsistency models and by comparing direct and indirect evidence (node splitting) on each node throughout the network, with *P*<0.05 indicating a significant difference^20–22^. In the NMA, the assumption of ‘consistency’ implies that estimates of treatment effects from direct and indirect evidence are in agreement, whereas evidence ‘inconsistency’ is the discrepancy between direct and indirect comparisons^23^. However, when direct and indirect comparisons do not exist simultaneously, the network diagram cannot form a closed loop, and no consistency test is required; a consistency fixed model is thus used. We used a random-effects model if significant heterogeneity existed; otherwise, we used a fixed-effects model. We used the surface under the cumulative ranking curve (SUCRA) with a larger area under the curve (0–100%) to rank the treatments for each outcome, indicating a better treatment measure. The transitivity assumption underlying the NMA was evaluated by comparing clinical and methodological variables distribution that could represent effect modifiers across the treatment comparisons.

## Results

### Literature searching

**Figure 1:**
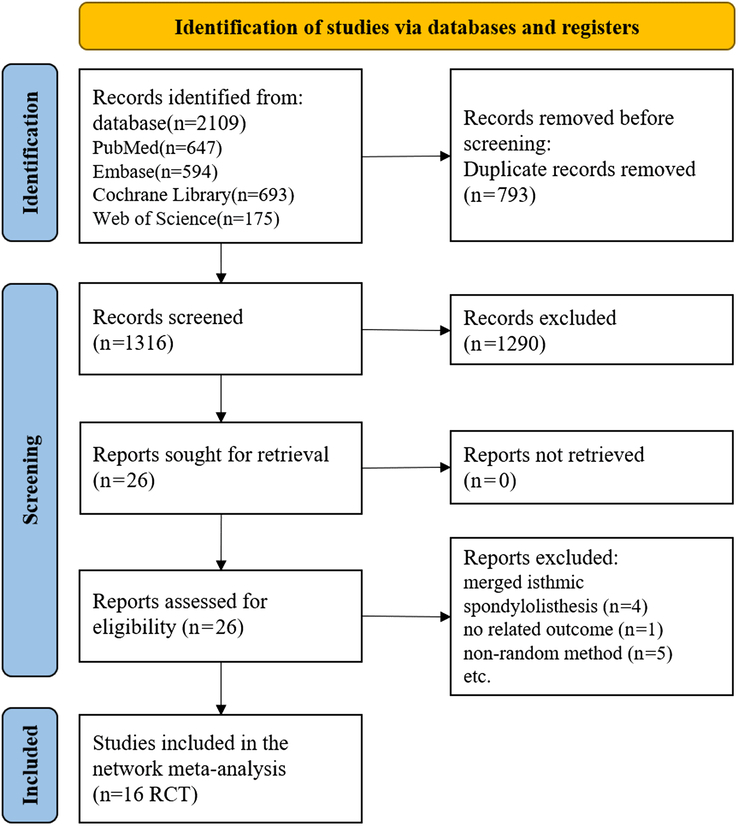
Flowchart of study selection and design.

Figure 1 shows the flowchart of the study selection process. We found 2109 references, comprising those from Embase (*n*=594), PubMed (*n*=647), the Cochrane Library (*n*=693), and Web of Science (*n*=175). Other references were identified by reviewing the reference lists of relevant studies. We used the ‘Find duplicates’ function in the EndNote X9 software (Thomson Corporation Corp, Stanford) to remove duplicates (*n*=793). After filtering the titles and abstracts, irrelevant references were excluded (*n*=1290), and the full text of the remaining references was retrieved (*n*=26). Finally, we included 16 RCTs that met the eligibility criteria.

### Characteristics and quality of included studies

**Figure 2:**
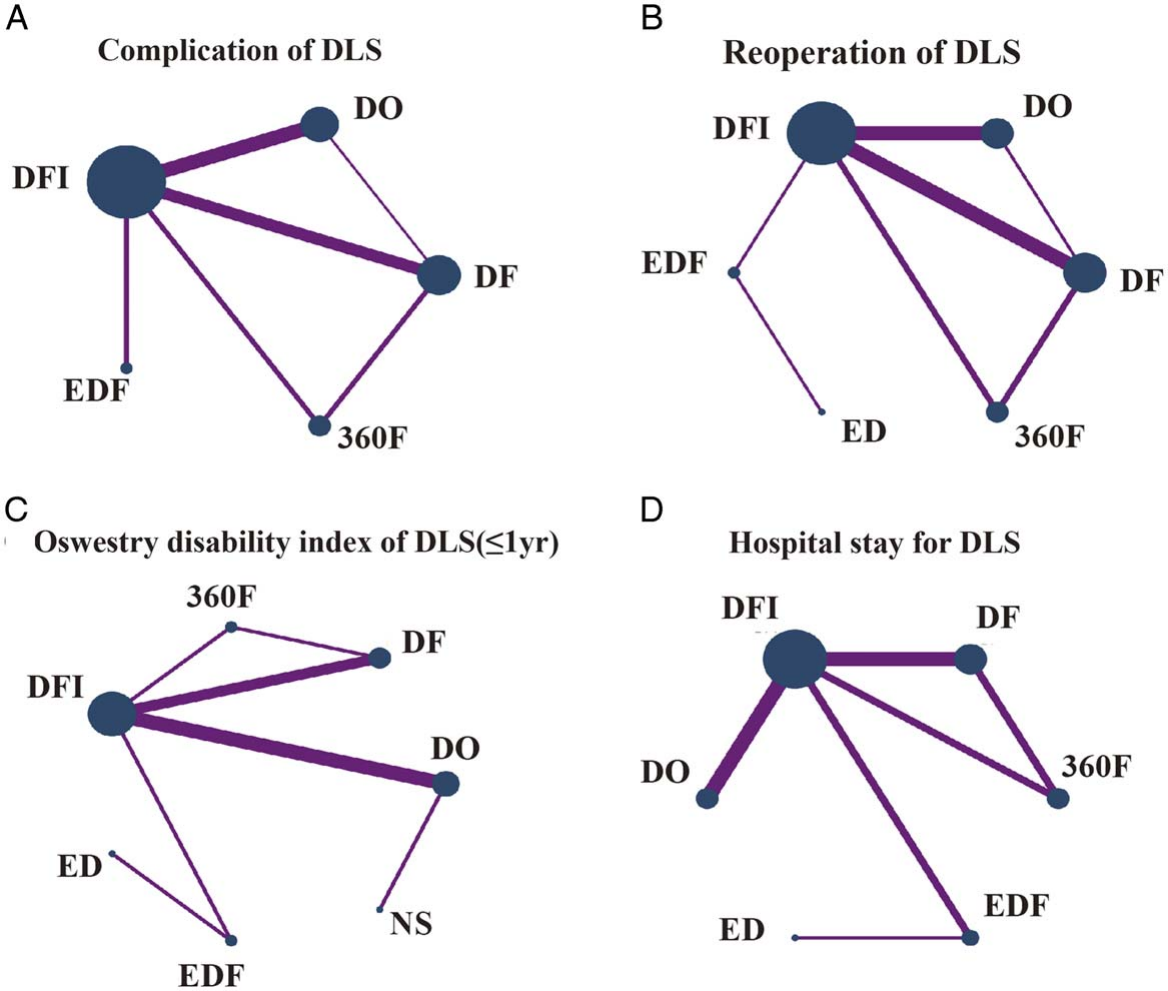
Network plots of comparison-based network meta-analyses in terms of complication (A), reoperation (B), short-term ODI (C) and hospital stay (D) for DLS. Each circular node represents a type of intervention. The circle size is proportional to the total number of patients. The width of the lines is proportional to the number of studies. performing head-to-head comparisons in the same study.

After screening via answering the questions included in the risk of bias tool (RoB2.0) excel worksheets, 16 trials involving 3273 patients and evaluating seven interventions (NS, DO, DF, DFI, EDF, ED, and 360F) were included^24–39^. Table 1 listed the basic information of all included studies. The mean age of the patients was 65.0 years, and 68.2% (2233/3273) were female. The risk of bias and the quality of each study were illustrated in Figure S1 (Supplemental Digital Content 4, http://links.lww.com/JS9/C48). All the studies had low risk of bias. Figure 2A–D and Figure S3a–g (Supplemental Digital Content 5, http://links.lww.com/JS9/C49) shows the NMA, which comprises 16 RCTs that evaluated seven intervention types. Figure 5A–D and Figure S5 a–g (Supplemental Digital Content 6, http://links.lww.com/JS9/C50) shows the predicted 95% CI comparing for different interventions.

**Table 1:** Characteristics of the included randomized controlled trials.

Study	Aria	Follow-up (months)	Interventions	Mean age	Male/female
Ghogawala *et al*. 2016^24^	USA	48	DO vs DFI	66.6	13/53
Bridwell *et al*. 1993^25^	USA	48	DO vs DFI vs DF	66.2	10/33
Austevoll *et al*. 2016^26^	Norway	12	DO vs DFI	65.6	190/507
Ghogawala *et al*. 2004^27^	USA	12	DO vs DFI	68.8	11/23
Mukai *et al*. 2013^28^	Japan	12	DFI vs EDF	65.4	8/32
Sembrano *et al*. 2016^29^	USA	24	DFI vs EDF	63.5	24/31
Pearson *et al*. 2011^30^	Lebanon	24	NS vs DO	66.1	185/406
Chan *et al*. 2019^31^	USA	24	EDF vs ED	67.2	64/79
Austevoll *et al*. 2021^32^	Norway	24	DO vs DFI	66.2	80/180
Cui *et al*. 2021^33^	China	24	DFI vs EDF	52.7	38/10
Davis *et al*. 2013^34^	USA	24	DF vs DFI	64.0	60/90
Abdu *et al*. 2018^35^	USA	96	NS vs DF vs DFI vs 360F	65.1	178/390
Abdu *et al*. 2009^36^	USA	48	DF vs DFI vs 360F	63.9	112/244
Inose *et al*. 2022^37^	Japan	147	DO vs DFI	63.4	28/32
Inose *et al*. 2018^38^	Japan	60	DO vs DFI	63.4	28/32
Andresen *et al*. 2023^39^	Denmark	24	DF vs DFI	71.5	11/91

360F, circumferential fusion; DF, decompression plus fusion without internal fixation; DFI, decompression plus fusion with internal fixation; DO, decompression only; ED, endoscopic decompression; EDF, endoscopic decompression plus fusion; NS, nonsurgery.

### Primary outcomes

#### ODI-based NMA

**Short-term outcomes (≤1-year):** Data from 16 RCTs involving 3273 participants were used to compare the ODI changes across different interventions^24–39^. NS treatments yielded a higher ODI score than surgical interventions, with a significant difference in the consistency model (Fig. S2a, Supplemental Digital Content 7, http://links.lww.com/JS9/C51). Based on the consistency model, the detected inconsistency in the NMA of the ODI scoring was insignificant; node-splitting analysis and heterogeneity showed insignificant inconsistency (*I* ^2^=12%, *P*=0.99, all *P*>0.05; Table S1–2, Supplemental Digital Content 8, http://links.lww.com/JS9/C52).

**Figure 3:**
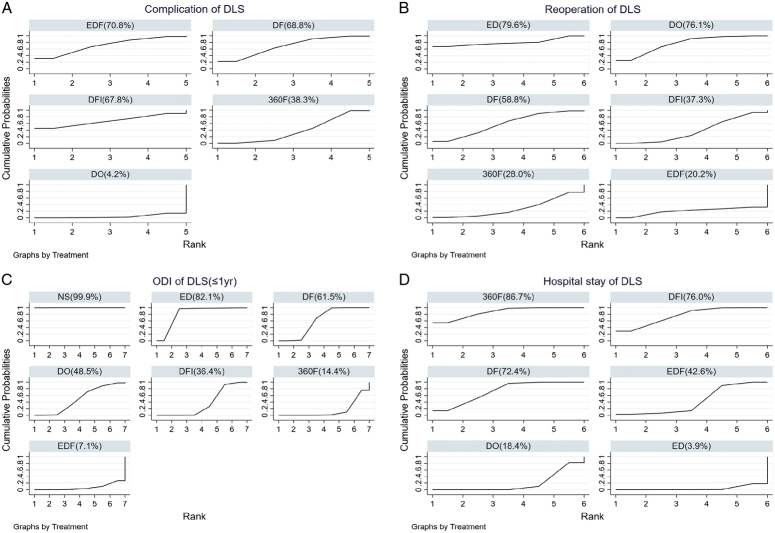
The SUCRA plots of different interventions to rank probability and ascertain the efficacies in terms of complication (A), reoperation (B), short-term ODI (C) and hospital stay (D) for DLS. The larger the SUCRA value, the larger the value of outcome indicator. SUCRA, surface under the cumulative ranking.

The network diagram is shown in Figure 2C. According to the SUCRA probability results (Fig. 3C), EDF improves the ODI in patients with DLS the most. The ranking of the seven interventions was as follows: NS (99.9%)>ED (82.1%)>DF (61.5%)>DO (48.5%)>DFI (36.4%)>360F (14.4%)>EDF (7.1%).

**Long-term outcomes (≥2**-**year):** Similarly, the network diagram is shown in Figure 3C and there was a significant difference in the superiority of surgical treatment over NS in improving the long-term ODI of patients. Based on the consistency model, the ranking of the six interventions was as follows (Fig. S4c, Supplemental Digital Content 9, http://links.lww.com/JS9/C53): NS (100.0%)>DO (69.4%)>DFI (48.9%)>DF (35.3%)>360F (25.4%)>EDF (21.0%) (*I* ^2^=8%, *P*=0.69, all *P*>0.05; Table S3–4, Supplemental Digital Content 8, http://links.lww.com/JS9/C52).

#### Short Form-36 (SF-36)

**Figure 4:**
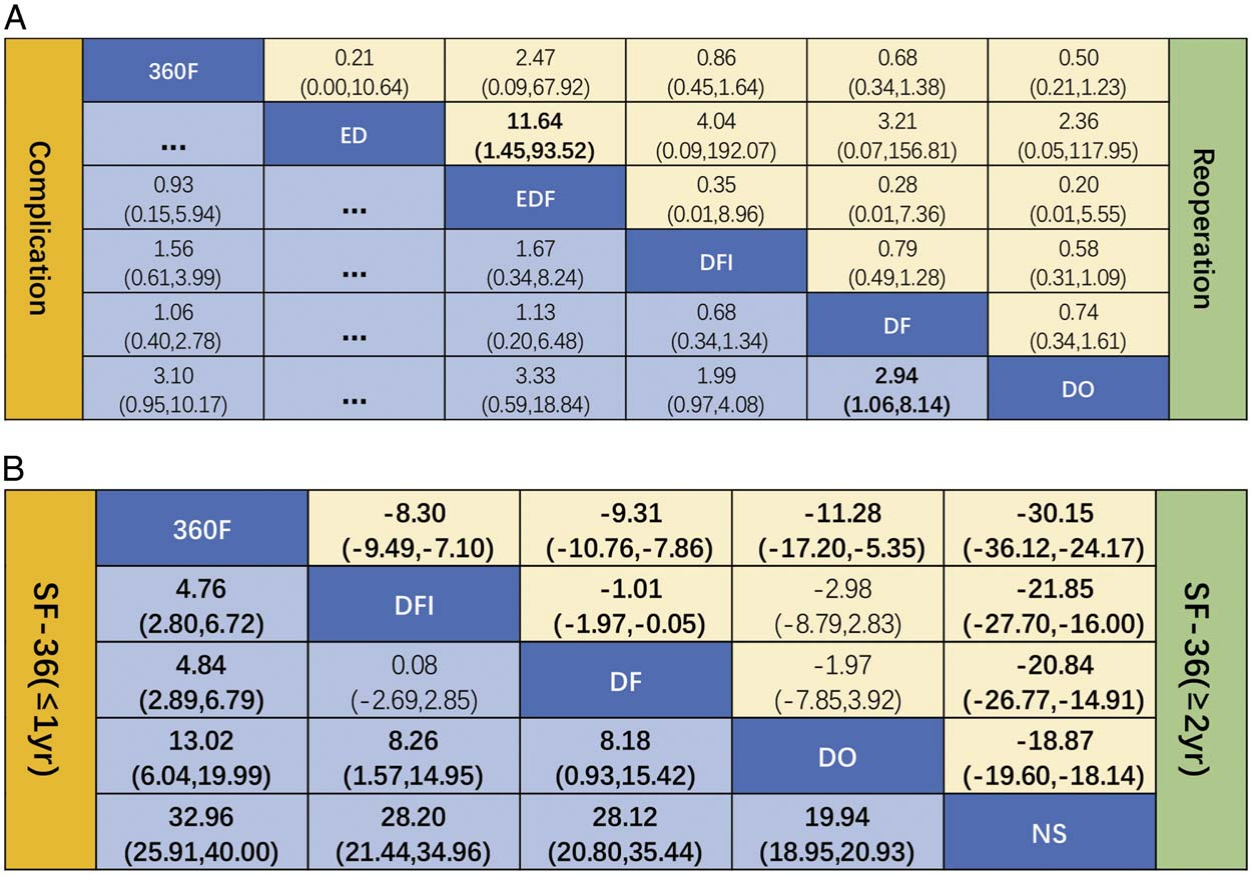
Complication (A), reoperation (A) and SF-36 (B) based network meta-analysis (NMA) in the consistency model. Data are ORs (95% CIs) in the column-defining treatment compared with the row-defining treatment; significant results are in bold. OR, odds ratio; Cis, credible intervals; SF-36, short form-36.

**Short-term outcomes (≤1 year):** Five RCTs reported data on the change in SF-36 for 1135 participants^27,30,34,36,39^. Surgical interventions were better than those of NS in relieving short and long-term SF-36 scores (Fig. S4e–f, Supplemental Digital Content 9, http://links.lww.com/JS9/C53). A significant difference was detected between any two interventions in the short-term and long-term SF-36 scores in the consistency model except for DFI versus DF, DFI versus DO, and DF versus DO (Fig. 4B). The heterogeneities and consistency for the short-term and long-term SF-36 were *I* ^2^=8%, *I* ^2^=7%, respectively (*P*=0.23) (Table S5–7, Supplemental Digital Content 8, http://links.lww.com/JS9/C52).

The rank possibility of the SF-36-based NMA in the consistency model ranged as follows (Fig. S4e, Supplemental Digital Content 9, http://links.lww.com/JS9/C53): 360F (100%)>DF (61.8%)>DFI (62.6%)>DO (25.6%)>NS (0.0%).

**Long-term outcomes (≥2 year):** For DLS treatment, 360F contributed to the highest long-term SF-36 score, while the lowest score was observed in NS. The SUCRA probability results were as follows (Fig. S4f, Supplemental Digital Content 9, http://links.lww.com/JS9/C53): 360F (75.0%)>DFI (46.0%)>DF (25.0%)>DO (19.4%)>NS (9.6%).

#### Low back pain evaluated using VAS of back pain-based NMA

Five RCTs reported VAS of back pain from 353 participants^28,33,34,37,39^, and the network diagram had no closed loops (Fig. S3g, Supplemental Digital Content 5, http://links.lww.com/JS9/C49) because the available data did not form loops. Based on the consistency fixed-effect model, insignificant differences were observed in the VAS score for back pain between any two surgical interventions (Fig. S2c, Supplemental Digital Content 7, http://links.lww.com/JS9/C51). The heterogeneity among the included studies differed insignificantly (*I* ^2^=0.1%, Table S8, Supplemental Digital Content 8, http://links.lww.com/JS9/C52). The ranking of the four interventions was as follows: (Fig. S4d, Supplemental Digital Content 9, http://links.lww.com/JS9/C53): DF (73.8%)>EDF (61.5%)>DFI (42.7%)>DO (22.0%).

### Secondary outcomes

#### Complication and reoperation rate-based NMA

**Figure 5:**
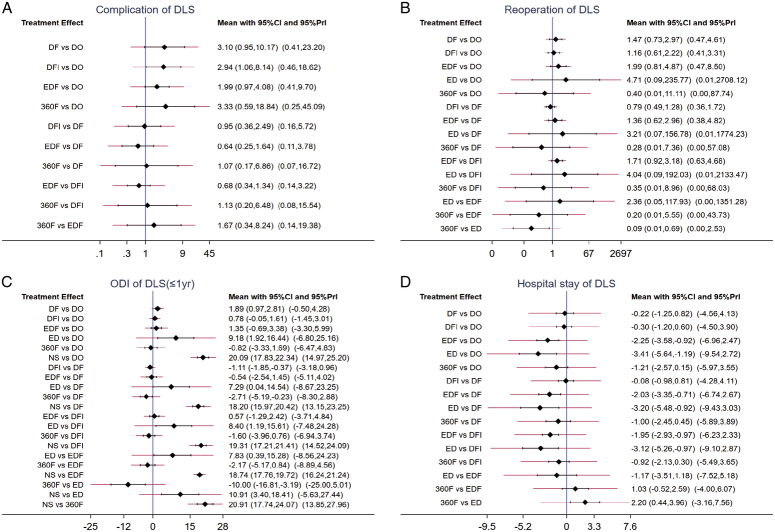
Forest plot for complication (A), reoperation (B), short-term ODI (C) and hospital stay (D) from network meta-analysis of mixed comparisons. Covering OR or WMD values, 95% CIs, 95% Prl. OR, odds ratio; Cis, credible intervals; Prl, prediction intervals; WMD, weighted mean difference.

Twelve RCTs with 2642 participants included complication and reoperation rates for different interventions^24,25,27,29–39^. The network diagram is shown in Figure 2A–B. Although EDF had a high complication rate, the reoperation rate was the lowest (Fig. 3a–b). In the consistent model, the mean difference in the reoperation and complication rates between any two surgical interventions was insignificant (Fig. 4A–B); for example, 360F versus EDF [odds ratio (OR) 0.93, 95% CI: 0.15–5.94], EDF versus DFI (OR 1.67, 95% CI: 0.34–8.24). The heterogeneity and consistency of the complications (*I* ^2^=6%, *P*=0.88) and reoperation (*I* ^2^=14%, *P*=0.84) are presented in Table S9–12 (Supplemental Digital Content 8, http://links.lww.com/JS9/C52). Node-splitting analysis revealed insignificant inconsistencies (all *P*>0.05). Figure 5A–B shows the OR and predicted 95% CI between any two interventions for complication and reoperation rates.

The assessment of the rank probabilities using SUCRA plots indicated that EDF had the highest potential to cause complications but the lowest reoperation rate. Among all the interventions to treat DLS, ED had the highest reoperation rate (Fig. 3A–B). The ranking of complications in five interventions was as follows: EDF (70.8%)>DF (68.8%)>DFI (67.8%)>360F (38.3%)>DO (4.2%). The ranking of reoperation rates in these interventions was as follows: ED (79.6%)>DO (76.1%)>DF (58.8%)>DFI (37.3%)>360F (28.0%)>EDF (20.2%).

#### Hospital stay-based NMA

Hospitalization data were reported for 1808 patients from 11 RCTs^24,29,31–39^. Decompression alone, including ED and DO, had the shortest hospitalization stay of all the interventions, with insignificant differences between interventions in a consistent model (Fig. S2b, Supplemental Digital Content 7, http://links.lww.com/JS9/C51). Figure 5D shows the predicted 95% CI comparing the length of hospital stay for different interventions. The network diagram is shown in Figure 2D (*I* ^2^=3%, *P*=0.21 Table S13–14, Supplemental Digital Content 8, http://links.lww.com/JS9/C52).

According to the SUCRA probability results (Fig. 3D), 360F might have had the longest hospital stay. The six interventions were ranked as follows: 360F (86.7%)>DFI (76.0%)>DF (72.4%)>EDF (42.6%)>DO (18.4%)>ED (3.9%).

#### Blood loss and operation time-based NMA

Twelve RCTs reported blood loss and operation time in 1908 participants^24,28,29,31–39^. Blood loss from EDF was higher than from other surgical operations, and DO appeared to have the minimum (Fig. S4a, Supplemental Digital Content 9, http://links.lww.com/JS9/C53). In addition, the ED procedure required less time than other interventions. The interventions differed insignificantly in the consistency random-effect model (Fig. S2d, Supplemental Digital Content 7, http://links.lww.com/JS9/C51), except when compared to 360F in respect of operation time (Fig. S2b, Supplemental Digital Content 7, http://links.lww.com/JS9/C51). The blood loss and operation time heterogeneities were *I* ^2^=6 and 3%, respectively, (*P*=0.12) (Table S15–17, Supplemental Digital Content 8, http://links.lww.com/JS9/C52). However, inconsistencies were identified in the operation time (*P*<0.05) (Table S18, Supplemental Digital Content 8, http://links.lww.com/JS9/C52). A possible reason for this result is that the data were extracted using a conversion formula, as the SD value was unavailable in all included studies^40^.

Based on the SUCRA probability results (Fig. S4a, Supplemental Digital Content 9, http://links.lww.com/JS9/C53), the ranking possibility of blood loss-based NMA in the consistency fixed model was as follows: EDF (82.5%)>DFI (63.9%)>360F (59.0%)>DF (51.2%)>ED (42.3%)>DO (1.1%). As for operation time, the ranking possibility was as follows (Fig. S4b, Supplemental Digital Content 9, http://links.lww.com/JS9/C53): 360F (99.3%)>EDF (68.1%)>DFI (58.9%)>DF (53.1%)>DO (11.2%)>ED (9.4%). The network diagram is shown in Figure S3a–b (Supplemental Digital Content 5, http://links.lww.com/JS9/C49).

#### Satisfaction-based NMA

Four RCTs reported satisfaction data from 938 participants^25,26,29,31^. The difference was insignificant regarding the satisfaction between any two interventions in the consistency fixed model (Fig. S2c, Supplemental Digital Content 7, http://links.lww.com/JS9/C51). The network diagram showed no closed loops (Fig. S3d, Supplemental Digital Content 5, http://links.lww.com/JS9/C49); therefore, consistency testing was not performed. The heterogeneity of the satisfaction was *I* ^2^=13% (Table S19, Supplemental Digital Content 8, http://links.lww.com/JS9/C52). The SUCRA probability results showed that the rank possibility of satisfaction-based NMA in the consistency fixed-effect model was as follows: DF (92.5%)>DFI (85.3%)>DO (50.8%)>EDF (27.4%)>ED (16.7%) (Fig. S4g, Supplemental Digital Content 9, http://links.lww.com/JS9/C53).

## Discussion

DLS is a common spinal disease in older adults, and most patients prefer surgery after failed conservative treatment^41^. Similarly, DLS is a common reason for spine surgery. DLS severely limits lumbar mobility and reduces quality of life^42^. This NMA was based on 16 RCTs that compared the efficacy of a wide range of currently used interventions for DLS. Surgical interventions were always significantly better at improving disability and functional status than NS; however, surgery caused more complications than NS interventions. Additionally, of all surgical interventions, EDF had the greatest improvement in short-term and long-term dysfunction and had the lowest reoperation rate; however, it had the highest complication rate and blood loss.

In this meta-analysis, the observations were divided into short-term (≤1 year) and long-term (≥2 year) time points. Weinstein *et al*.^43^ found that surgical interventions had a significant efficacy for DLS at 3 months and 1 year; however, it showed a slightly decreasing trend over 2 years. Owing to incomplete data from some of the included RCTs, we prioritized data closest to the 1-year and 2-year time points, resulting in more adequate inclusion^44^.

Consistency is a prerequisite guarantee for analyzing the results of NMA. In this NMA, we analyzed all indicators for consistency and found inconsistency only in the indicator of operation time. One limitation was data incompleteness, such as SD and standard error not being reported in some of the studies^26,28,29,34,39^. Therefore, the formula was modified to fully refine the data; however, there are some inaccuracies in the results obtained by converting the formula^40^. Second, in two RCTs with multiarm (DFI, DF, and 360F) comparisons (Fig. S3b, Supplemental Digital Content 5, http://links.lww.com/JS9/C49), we analyzed consistency by the node-splitting method. We detected an inconsistency in the direct versus indirect comparisons (Table S18, Supplemental Digital Content 8, http://links.lww.com/JS9/C52). This local inconsistency might account for the overall heterogeneity in the operation time. To circumvent the causes of inconsistency, inconsistencies should be further assessed through subgroup analysis and meta-regression or by excluding relevant literature. However, because of the temporary lack of relevant data, subgroup analysis was not performed in this NMA.

NS treatment, including physical therapy, epidural injections, chiropractic treatment, anti-inflammatory agents, and opioid analgesic agents, is preferred for patients with less severe DLS symptoms. The effectiveness of surgical compared with NS interventions has not been demonstrated^43^. Therefore, we investigated vital outcomes, such as disability, pain, complications, reoperation, operation time, blood loss, and hospitalization, to evaluate the effects of all available interventions. In this NMA study, all surgical methods showed satisfactory clinical results, especially in DF and DFI. However, endoscopic surgery has been reported in the literature to be satisfactory in improving short-term outcomes^45^. In our study, its lower satisfaction rate might have been because of the increased surgical cost, which should be further analyzed by NMA in the future as data related to surgical cost is often reported in the literature.

In the past few decades, new surgical approaches and the development of optical technology have expanded the scope of lumbar endoscopic surgery^46,47^. The advantages of endoscopic surgery include enhanced recovery after surgery, less invasiveness, and lower reoperation rates. We found that EDF is less likely to be painful and dysfunctional owing to less damage to normal structures, relieves short-term and long-term pain effectively, has shorter hospitalization times, and lowered reoperation rates, making it a good choice for patients with DLS. However, longer operation time, unclear vision, and incomplete hemostasis due to undefined bleeding points might have led to increased bleeding in EDF. The increased complications associated with EDF included increased intraoperative bleeding, longer operation times, and a longer learning time for endoscopic procedures. Finally, multiple open surgical accesses exist, including anterior, transforaminal, and posterior lumbar interbody fusion, but only a single endoscopic surgical access. Therefore, there is a need to develop more endoscopic surgical accesses for more precise treatments.

Whether or not to perform fusion is a controversial issue in treating DLS, where Kaiser *et al*.^48^ found that DO was adequate compared with instrumented fusion. Nevertheless, we found that in open or endoscopic surgery, decompression with fusion (DF and DFI) was superior to DO in improving patient pain, dysfunction, and reoperation rates. However, DO was superior to fusion concerning complications, bleeding, and operative time.

**Figure 6:**
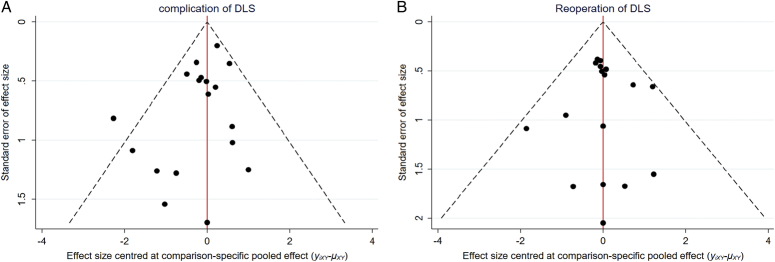
Funnel plot of complication (A) and reoperation (B) for DLS based network meta-analysis (NMA) in the consistency model.

Funnel plots were used to assess publication bias, which was roughly symmetrically distributed in this study, except for the operation time, blood loss, and length of hospital stay (Fig. S6a–i, Supplemental Digital Content 10, http://links.lww.com/JS9/C54), suggesting publication bias. Operation time bias could be because of the inconsistencies mentioned above. In terms of blood loss, bias might be associated with the measurement methods for intraoperative, postoperative, and occult blood loss. Finally, regarding the length of hospital stay, bias might have been related to the recording of preoperative, postoperative, and total times. Overall, the evaluation of publication bias in funnel plots is highly subjective. The points in the funnel plot represent the included RCTs, and publication bias was considered low if the points were roughly symmetrically distributed^49,50^ (Fig. 6C).

Although our NMA included all mainstream interventions to provide comprehensive results, this study has some limitations. First, only high-quality RCTs were included because we strictly adhered to the inclusion criteria and RoB2 Cochrane collaboration tools to screen the literature. This approach filtered out ‘poor quality’ studies and studies that included isthmic spondylolisthesis and no relevant results. Therefore, the sample size was small, reducing the statistical power of the analysis. In the future, we will incorporate the results of more ‘moderate quality’ or ‘lower quality’ RCTs for further analysis. Second, the integrating prognostic indicators were reported at different time points, possibly contributing to heterogeneity. Third, the data in the literature were incomplete, with no subgroup analysis of surgical segment location, follow-up time, number of segments operated on, type of slip, or refinement of patient characteristic types required for precision medicine. Significant heterogeneity of follow-up time was identified in this study, and subgroup analysis was not performed owing to the limited data.

This NMA compared the common treatment modalities for DLS. The ODI, SF-36, VAS of back pain, complications, reoperation rate, operative time, blood loss, hospital stay, and satisfaction were ranked. We found that EDF has some advantages for the treatment of DLS. At the same time, we discuss the advantages of endoscopic surgery, inconsistency in data, with or without fusion, and the shortcomings of this study. The results of this NMA can serve as a guide to the clinical management of DLS and for the development of novel therapeutic options.

## Conclusion

Surgical interventions were superior to NS interventions in relieving pain and dysfunction in DLS and had a higher complication rate. Among all operative approaches, EDF may be the optimal treatment option for DLS over others owing to its advantages in improving pain and dysfunction, lowering reoperation rates, and having a shorter hospital stay.

## Ethical approval and consent to participate

Not applicable.

## Consent

Not applicaple.

## Sources of funding

This study was supported by the National Natural Science Foundation of China (Grant No 82360430; 82360433).

## Author contribution

H.J. and Z.Z.: writing the original draft, reviewing and editing, methodology, and formal analysis; J.P.Q.: resources, methodology, and formal analysis; L.P.B.: resources, methodology, formal analysis, and data curation; H.Q.: resources, data curation, and supervision; J.A.: conceptualization, reviewing and editing the manuscript, formal analysis, and supervision. All authors read and approved the final manuscript.

## Conflicts of interest disclosure

The authors declare no conflicts of interest for this work.

## Research registration unique identifying number (UIN)

The PROSPERO number is CRD42022377470.

## Guarantor

Hu Qian, Department of Orthopedic Surgery, Affiliated Hospital of Zunyi Medical University, 149 Dalian Road, Zunyi 563000, People’s Republic of China. E-mail: moneylakecsu@163.com.

## Data availability statement

Data availability is not applicable to this article as no new data were created or analyzed in this study.

Availability of data and materials: All data and materials are available. The corresponding author can provide the datasets used and/or analyzed during the current study upon reasonable request.

## Provenance and peer review

Not applicaple.

## Supplementary Material

**Figure:**
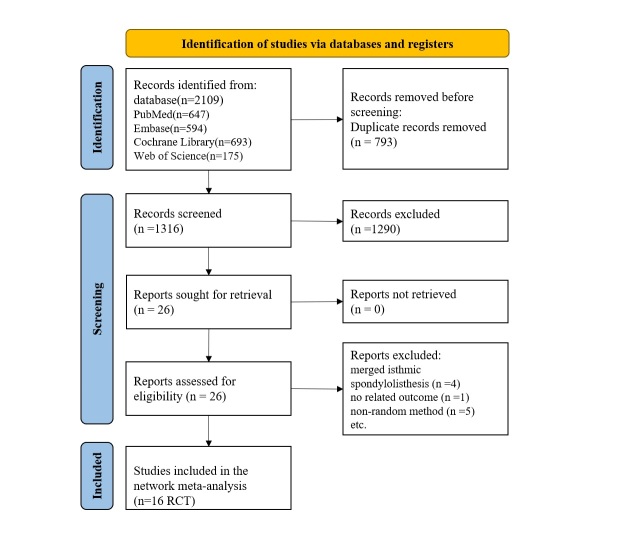


**Figure:**
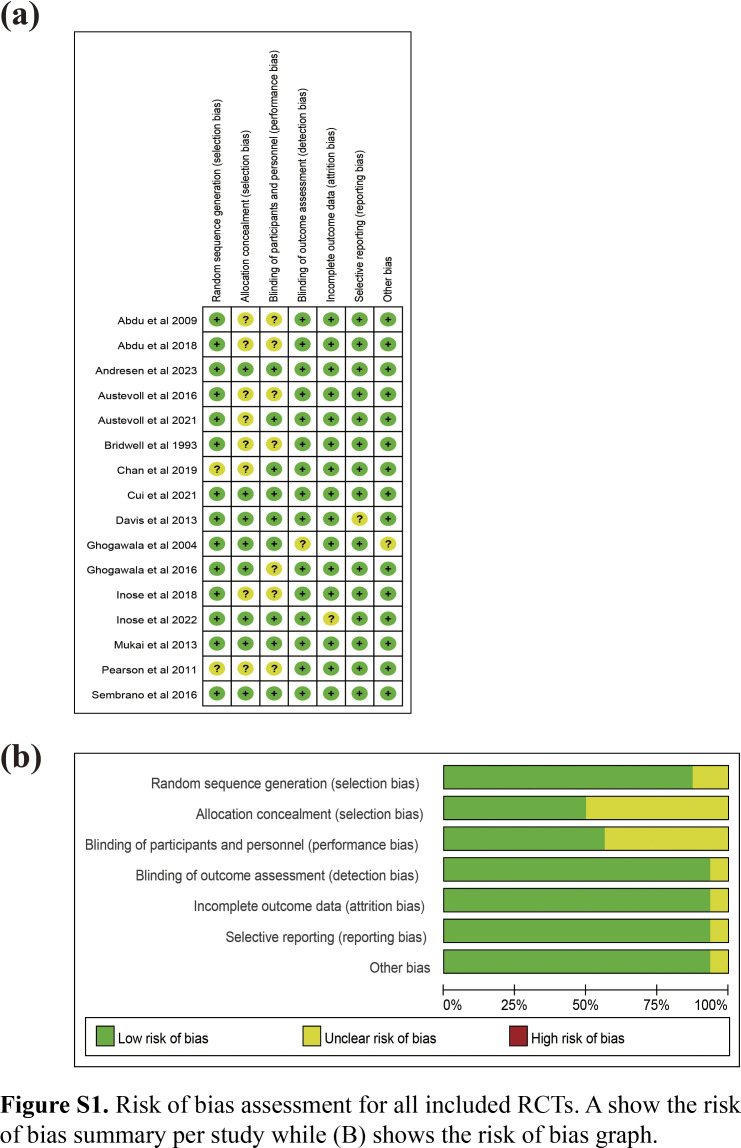


**Figure:**
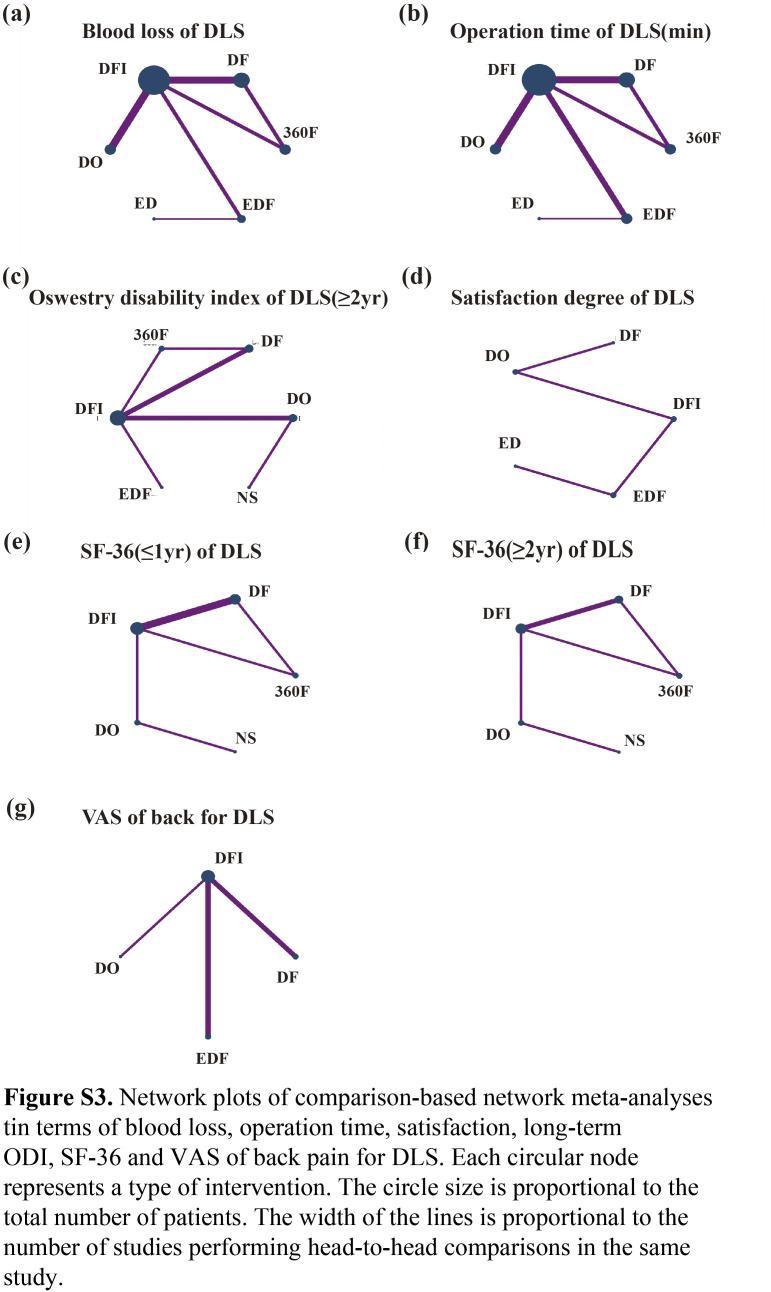


**Figure:**
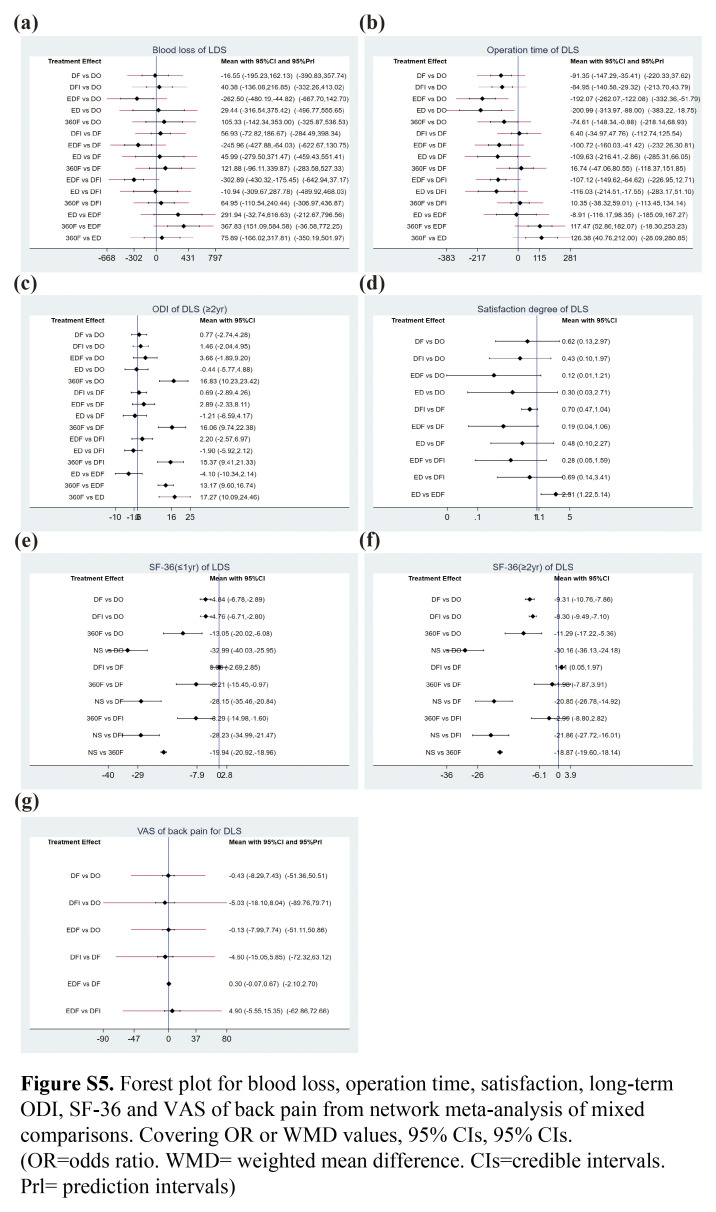


**Figure:**
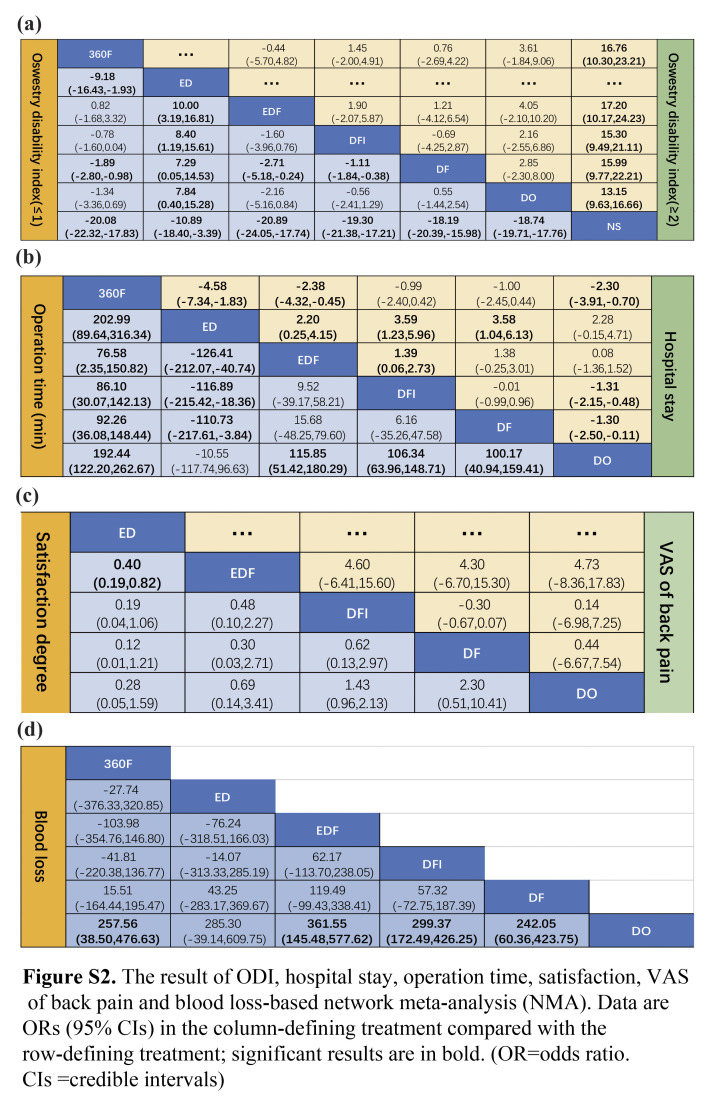


**Figure:**
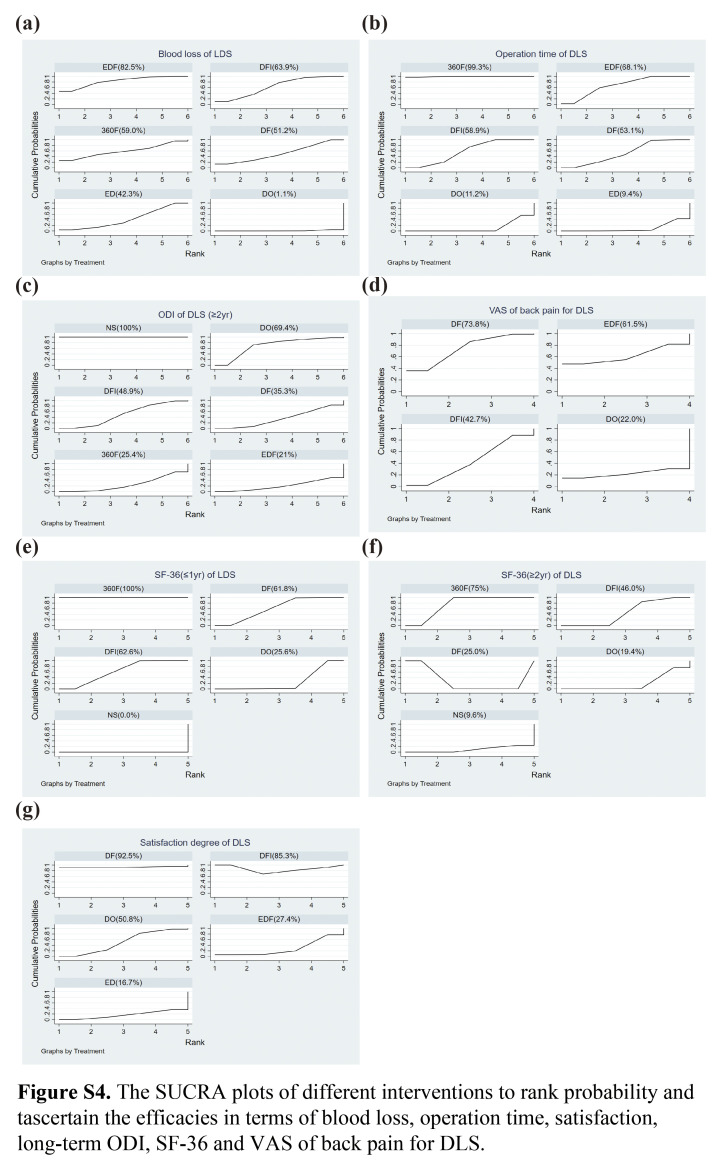


**Figure:**