# Supplementary material for: Management for degenerative lumbar spondylolisthesis: a network meta-analysis and systematic review basing on randomized controlled trials
Source: Int J Surg. 2024 Mar 4;110(5):3050–9. doi: 10.1097/JS9.0000000000001228 (PMC11093486; doi:10.1097/JS9.0000000000001228)
Supplement: Supplementary file 3 [file js9-110-3050-s008.docx]

**Table S1**

**ODI(≤1yr) of DLS for heterogeneity**

1. Empirical mean and standard deviation for each variable,

plus standard error of the mean:

Mean SD Naive SE Time-series SE

d.DFI.360F -0.7728 3.515 0.02485 0.03045

d.DFI.DF 1.1152 2.457 0.01737 0.02744

d.DFI.DO 1.1703 2.319 0.01640 0.04195

d.DFI.EDF -1.5924 3.918 0.02770 0.03472

d.DO.NS 18.7495 3.756 0.02656 0.02679

d.EDF.ED 10.1073 5.125 0.03624 0.10888

sd.d 2.8066 2.546 0.01800 0.12989

2. Quantiles for each variable:

2.5% 25% 50% 75% 97.5%

d.DFI.360F -8.4028 -1.92654 -0.7767 0.40282 6.584

d.DFI.DF -4.1671 0.15786 1.1123 2.09383 6.437

d.DFI.DO -2.7706 -0.07738 0.9618 2.12416 6.655

d.DFI.EDF -9.9212 -3.21102 -1.5470 -0.00737 6.654

d.DO.NS 10.9014 17.50034 18.7375 19.97055 26.906

d.EDF.ED 0.3240 7.09296 10.0075 13.06591 20.052

sd.d 0.1335 0.98557 2.0736 3.90023 9.624

-- Model fit (residual deviance):

Dbar pD DIC

22.59950 18.69125 41.29075

21 data points, ratio 1.076, I^2 = 12%

**Table S2**

**ODI(≤1yr) of DLS for consistency**

**Consistency of a loop**

**Consistency of node**

**Table S3**

**ODI(≥2yr) of DLS for heterogeneity**

1. Empirical mean and standard deviation for each variable,

plus standard error of the mean:

Mean SD Naive SE Time-series SE

d.DFI.360F -1.4017 5.300 0.03747 0.04385

d.DFI.DF -0.4369 4.202 0.02971 0.03876

d.DFI.DO 2.6976 4.467 0.03159 0.04866

d.DFI.EDF -1.8715 5.747 0.04064 0.04239

d.DO.NS 13.1126 5.655 0.03999 0.04108

sd.d 4.5767 3.283 0.02321 0.11391

2. Quantiles for each variable:

2.5% 25% 50% 75% 97.5%

d.DFI.360F -13.2641 -3.4595 -1.5124 0.7471 10.042

d.DFI.DF -9.3915 -2.1764 -0.7044 1.3851 9.022

d.DFI.DO -6.2768 0.4242 2.4257 4.7803 12.864

d.DFI.EDF -14.2174 -4.2308 -1.8472 0.5045 10.759

d.DO.NS 0.6415 10.9189 13.1678 15.3057 25.539

sd.d 0.2205 1.9393 3.8371 6.6655 12.099

-- Model fit (residual deviance):

Dbar pD DIC

13.01353 12.49498 25.50851

13 data points, ratio 1.001, I^2 = 8%

**Table S4**

**ODI(≥2yr) of DLS for consistency**

**Consistency of a loop**

**Consistency of node**

**Table S5**

**SF-36(≤1yr) of DLS for heterogeneity**

1. Empirical mean and standard deviation for each variable,

plus standard error of the mean:

Mean SD Naive SE Time-series SE

d.B.A -19.9355 7.752 0.05482 0.05511

d.B.D2 8.3496 8.437 0.05966 0.08845

d.D2.D1 -0.4644 5.651 0.03996 0.04884

d.D2.F 4.5787 7.432 0.05256 0.06182

sd.d 5.8006 5.289 0.03740 0.19418

2. Quantiles for each variable:

2.5% 25% 50% 75% 97.5%

d.B.A -37.2763 -22.115 -19.9452 -17.776 -2.138

d.B.D2 -10.5934 4.515 8.4163 12.021 26.785

d.D2.D1 -13.4957 -2.212 -0.2481 1.279 12.244

d.D2.F -12.5355 2.470 4.6604 6.709 21.310

sd.d 0.1183 1.544 3.9035 9.041 18.474

-- Model fit (residual deviance):

Dbar pD DIC

8.734674 8.697415 17.432089

9 data points, ratio 0.9705, I^2 = 8%

**Table S6**

**SF-36(≤1yr) of DLS for consistency**

**Consistency of a loop**

**Consistency of node**

**Table S7**

**SF-36(≥2yr) of DLS for heterogeneity**

1. Empirical mean and standard deviation for each variable,

plus standard error of the mean:

Mean SD Naive SE Time-series SE

d.DFI.360F 8.217 7.438 0.05260 0.06466

d.DFI.DF -1.125 5.732 0.04053 0.05013

d.DFI.DO -2.939 8.218 0.05811 0.09110

d.DO.NS -18.759 7.766 0.05491 0.05526

sd.d 5.803 5.117 0.03618 0.19369

2. Quantiles for each variable:

2.5% 25% 50% 75% 97.5%

d.DFI.360F -8.7860 6.005 8.277 10.3589 25.0146

d.DFI.DF -14.1164 -2.938 -1.101 0.5735 12.0586

d.DFI.DO -20.7759 -6.600 -3.066 0.6581 15.5958

d.DO.NS -36.2835 -20.990 -18.823 -16.5180 -0.8198

sd.d 0.1659 1.541 4.150 9.1183 17.6134

-- Model fit (residual deviance):

Dbar pD DIC

8.627273 8.616838 17.244111

9 data points, ratio 0.9586, I^2 = 7%

**Table S8**

**VAS of back pain for DLS’ heterogeneity**

1. Empirical mean and standard deviation for each variable,

plus standard error of the mean:

Mean SD Naive SE Time-series SE

d.DFI.DF 0.4128 4.102 0.029006 0.11680

d.DFI.DO -4.6147 5.868 0.041490 0.16096

d.DFI.EDF 0.4277 2.632 0.018612 0.03250

sd.d 2.3257 1.393 0.009853 0.04712

2. Quantiles for each variable:

2.5% 25% 50% 75% 97.5%

d.DFI.DF -7.34704 -2.3271 0.3684 3.1379 8.837

d.DFI.DO -15.95337 -8.5728 -4.5674 -0.5757 6.863

d.DFI.EDF -5.13622 -0.7543 0.3597 1.6033 6.235

sd.d 0.09886 1.1277 2.2869 3.5202 4.663

-- Model fit (residual deviance):

Dbar pD DIC

9.011576 8.167688 17.179264

10 data points, ratio 0.9012, I^2 = 0.1%

**Table S9**

**Complication of DLS for heterogeneity**

1. Empirical mean and standard deviation for each variable,

plus standard error of the mean:

Mean SD Naive SE Time-series SE

d.DFI.360F 0.6151 0.6283 0.004442 0.009467

d.DFI.DF 0.5293 0.4496 0.003179 0.006794

d.DFI.DO -0.9159 0.4615 0.003263 0.008102

d.DFI.EDF 0.0235 0.8634 0.006105 0.016024

sd.d 0.7526 0.3213 0.002272 0.010126

2. Quantiles for each variable:

2.5% 25% 50% 75% 97.5%

d.DFI.360F -0.6383 0.2304 0.61073 0.9897 1.88625

d.DFI.DF -0.3169 0.2443 0.51073 0.7935 1.47548

d.DFI.DO -1.9298 -1.1891 -0.88261 -0.6094 -0.09864

d.DFI.EDF -1.6929 -0.5214 0.02466 0.5827 1.70890

sd.d 0.2443 0.5267 0.70504 0.9321 1.51657

-- Model fit (residual deviance):

Dbar pD DIC

27.52346 20.37404 47.89750

27 data points, ratio 1.019, I^2 = 6%

**Table S10**

**Complication of DLS for consistency**

** Consistency of a loop**

**Consistency of node**

**Table S11**

**Reoperation of DLS for heterogeneity**

1. Empirical mean and standard deviation for each variable,

plus standard error of the mean:

Mean SD Naive SE

d.DFI.360F -0.009687 0.4866 0.003441

d.DFI.DF 0.353830 0.3644 0.002577

d.DFI.DO 0.321003 0.4120 0.002913

d.DFI.EDF -25.504308 17.1195 0.121053

d.EDF.ED 3.138262 1.4439 0.010210

sd.d 0.451128 0.3577 0.002530

Time-series SE

d.DFI.360F 0.012964

d.DFI.DF 0.008607

d.DFI.DO 0.010893

d.DFI.EDF 2.874893

d.EDF.ED 0.068765

sd.d 0.020148

2. Quantiles for each variable:

2.5% 25% 50% 75%

d.DFI.360F -0.975807 -0.28991 -0.002622 0.2740

d.DFI.DF -0.322549 0.14725 0.326017 0.5401

d.DFI.DO -0.532178 0.08009 0.328082 0.5601

d.DFI.EDF -59.277656 -37.52717 -24.505188 -9.9910

d.EDF.ED 0.707011 2.09336 2.990208 4.1018

sd.d 0.005746 0.17231 0.376403 0.6427

97.5%

d.DFI.360F 0.9772

d.DFI.DF 1.1698

d.DFI.DO 1.1310

d.DFI.EDF -1.4401

d.EDF.ED 6.1458

sd.d 1.3581

-- Model fit (residual deviance):

Dbar pD DIC

27.74649 15.95365 43.70015

25 data points, ratio 1.11, I^2 = 14%

**Table S12**

**Reoperation of DLS for consistency**

**Consistency of a loop**

**Consistency of node**

**Table S13**

**Hospital stay of DLS for heterogeneity**

1. Empirical mean and standard deviation for each variable,

plus standard error of the mean:

Mean SD Naive SE Time-series SE

d.DFI.360F 1.0731 0.7884 0.005575 0.011635

d.DFI.DF 0.1853 0.5664 0.004005 0.005029

d.DFI.DO -1.9592 0.5889 0.004164 0.005882

d.DFI.EDF -1.1763 0.8104 0.005730 0.007170

d.EDF.ED -2.2028 1.1097 0.007847 0.008399

sd.d 1.0198 0.3478 0.002459 0.006424

2. Quantiles for each variable:

2.5% 25% 50% 75% 97.5%

d.DFI.360F -0.4861 0.5829 1.0738 1.5604 2.65707

d.DFI.DF -0.9591 -0.1557 0.1915 0.5314 1.29081

d.DFI.DO -3.1813 -2.3154 -1.9471 -1.5959 -0.81460

d.DFI.EDF -2.8889 -1.6467 -1.1461 -0.6688 0.38983

d.EDF.ED -4.4169 -2.8717 -2.1985 -1.5429 0.01907

sd.d 0.5297 0.7744 0.9518 1.1948 1.90259

-- Model fit (residual deviance):

Dbar pD DIC

23.68669 22.77739 46.46408

24 data points, ratio 0.9869, I^2 = 3%

**Table S14**

**Hospital stay of DLS for consistency**

**Consistency of a loop**

**Consistency of node**

**Table S15**

**Blood loss of DLS for heterogeneity**

1. Empirical mean and standard deviation for each variable,

plus standard error of the mean:

Mean SD Naive SE Time-series SE

d.DFI.360F -42.81 111.64 0.7894 1.1820

d.DFI.DF -56.06 81.69 0.5776 0.7268

d.DFI.DO -303.69 81.73 0.5779 0.9471

d.DFI.EDF 64.62 114.32 0.8084 0.8631

d.EDF.ED -77.51 159.48 1.1277 1.1370

sd.d 149.27 51.71 0.3656 1.0294

2. Quantiles for each variable:

2.5% 25% 50% 75% 97.5%

d.DFI.360F -267.60 -110.913 -44.23 25.780 181.6

d.DFI.DF -218.26 -107.061 -56.50 -6.535 107.4

d.DFI.DO -471.94 -351.604 -302.84 -254.525 -142.0

d.DFI.EDF -169.97 -3.553 65.83 134.027 290.9

d.EDF.ED -402.82 -169.929 -76.79 16.577 239.3

sd.d 77.29 112.983 139.67 174.233 277.8

-- Model fit (residual deviance):

Dbar pD DIC

24.36039 23.33777 47.69817

24 data points, ratio 1.015, I^2 = 6%

**Table S16**

**Blood loss of DLS for consistency**

**Consistency of a loop**

**Consistency of node**

**Table S17**

**Operation time of DLS for heterogeneity**

1. Empirical mean and standard deviation for each variable,

plus standard error of the mean:

Mean SD Naive SE Time-series SE

d.DFI.360F 87.309 34.89 0.2467 0.3222

d.DFI.DF -5.877 25.91 0.1832 0.2168

d.DFI.DO -107.267 26.75 0.1891 0.2142

d.DFI.EDF 9.803 30.34 0.2145 0.2326

d.EDF.ED -125.605 53.10 0.3755 0.4136

sd.d 48.986 14.98 0.1060 0.2630

2. Quantiles for each variable:

2.5% 25% 50% 75% 97.5%

d.DFI.360F 18.39 65.861 86.991 108.56 157.42

d.DFI.DF -57.13 -21.952 -6.017 10.16 45.99

d.DFI.DO -160.93 -123.370 -107.340 -91.15 -54.17

d.DFI.EDF -50.57 -8.945 9.920 28.71 70.33

d.EDF.ED -231.17 -158.358 -126.055 -93.44 -16.78

sd.d 28.76 38.673 46.140 55.99 86.82

-- Model fit (residual deviance):

Dbar pD DIC

25.83616 25.38387 51.22003

26 data points, ratio 0.9937, I^2 = 3%

**Table S18**

**Operation time of DLS for consistency**

**Consistency of a loop**

**Consistency of node**

**Table S19**

**Satisfaction degree of DLS for heterogeneity**

1. Empirical mean and standard deviation for each variable,

plus standard error of the mean:

Mean SD Naive SE Time-series SE

d.DFI.DO -0.2177 0.3480 0.002461 0.003479

d.DFI.EDF -0.2419 0.4746 0.003356 0.009143

d.DO.DF 0.8099 0.7256 0.005131 0.021149

d.EDF.ED -0.5480 0.3987 0.002819 0.006071

sd.d 0.2820 0.1648 0.001165 0.004621

2. Quantiles for each variable:

2.5% 25% 50% 75% 97.5%

d.DFI.DO -0.9521 -0.3990 -0.2164 -0.03653 0.5259

d.DFI.EDF -1.1898 -0.5380 -0.2443 0.05330 0.7232

d.DO.DF -0.5013 0.3089 0.7560 1.27095 2.3570

d.EDF.ED -1.3622 -0.7819 -0.5461 -0.31576 0.2793

sd.d 0.0146 0.1401 0.2775 0.42426 0.5581

-- Model fit (residual deviance):

Dbar pD DIC

8.016158 7.761445 15.777603

8 data points, ratio 1.002, I^2 = 13%
